# Hyaluronan Is Not a Ligand but a Regulator of Toll-Like Receptor Signaling in Mesangial Cells: Role of Extracellular Matrix in Innate Immunity

**DOI:** 10.1155/2014/714081

**Published:** 2014-01-21

**Authors:** Rainer Ebid, Julia Lichtnekert, Hans-Joachim Anders

**Affiliations:** Nephrologisches Zentrum, Medizinische Klinik und Poliklinik IV der LMU, Pettenkoferstr. 8a, 80336 Munich, Germany

## Abstract

Glomerular mesangial cells (MC), like most cell types secrete hyaluronan (HA), which attached to the cell surface via CD44, is the backbone of a hydrophilic gel matrix around these cells. Reduced extracellular matrix thickness and viscosity result from HA cleavage during inflammation. HA fragments were reported to trigger innate immunity via Toll-like receptor-(TLR-) 2 and/or TLR4 in immune cells. We questioned whether HA fragments also regulate the immunostimulatory capacity of smooth muscle cell-like MC. LPS (TLR4-ligand) and PAM3CysSK4 (TLR2-ligand) induced IL-6 secretion in MC; highly purified endotoxin-free HA < 3000 Da up to 50 **μ**g/mL did not. Bovine-testis-hyaluronidase from was used to digest MC-HA into HA fragments of different size directly in the cell culture. Resultant HA fragments did not activate TLR4-deficient MC, while TLR2-deficient MC responded to LPS-contamination of hyaluronidase, not to produced HA fragments. Hyaluronidase increased the stimulatory effect of TLR2-/-3/-5 ligands on their TLR-receptors in TLR4-deficient MC, excluding any effect by LPS-contamination. Supplemented heparin suppressed every stimulatory effect in a dose-dependent manner. We conclude that the glycosaminoglycan HA creates a pericellular jelly barrier, which covers surface receptors like the TLRs. Barrier-thickness and viscosity balanced by HA-synthesis and degradation and the amount of HA-receptors on the cell surface regulate innate immunity via the accessibility of the receptors.

## 1. Background

In 1934, Meyer and Palmer were the first to describe hyaluronan (HA, formerly called hyaluronic acid) as an isolate from the vitreous humor [1]. Since then, HA has been extensively examined in terms of its physical, chemical, and biological properties [2, 3]. HA occurs ubiquitously throughout the body, serving as the backbone of hydrate coat surrounding cells [4, 5], when attached to the cell surface. The main receptor on the cell surface is CD44 [6]. HA of high-molecular weight (4 × 10^5^–2 × 10^7^ Da[lton]) can bind water in a relation 1 : 1000 [2]. Degradation of HA reduces the binding capacity for water and turns the aqueous gel into a fluid of low viscosity [7]. HA occurs in viscous fluids such as the synovial fluid, which is the rationale for its topical use in diseases of the joint [8]. High-molecular weight HA is also used topically in aesthetic medicine [9] while, a preparation for systemic application has been used in horses and greyhounds [10, 11]. Another aspect of HA is its immunologic effect. Termeer et al. identified HA as an agonist for Toll-like receptor-(TLR-) 4 [12]. In contrast, Scheibner et al. described HA as an agonist for TLR2 and disproved TLR4 agonistic activity [4]. TLRs are one of the families of innate pattern recognition receptors that can trigger nuclear factor (NF)-*κ*B activation by outside-in signaling [13]. TLR2 and TLR4 were first described to recognize bacterial lipopeptides and lipopolysaccharides (LPS) from Gram-positive and Gram-negative bacteria [14, 15]. TLR2 and TLR4 both reside on the cell surface while nucleic acid-specific TLRs occur on intracellular endosomes [13]. Later, it was found that also endogenous molecules bind to TLR2 and TLR4 which allows tissues to recognize also noninfectious types of injury and to translate this into local inflammation [16].

This process of “danger signalling” is crucial in understanding kidney diseases because of their mostly sterile nature [17]. In fact, TLRs and other pattern recognition receptors contribute to the immunopathology of the kidney by initiating intrarenal inflammation, leukocyte recruitment, and unnecessary tissue damage [18–20]. This particularly applies to the glomerular compartment of the kidney which filters the blood and is therefore exposed to all circulating elements. For example, circulating agonists to all known TLRs have the potential to aggravate a preexisting glomerulonephritis, for example, by activating immune cells inside and outside the glomerulus [21–24]. However, also intrinsic renal cells express TLRs; for example, glomerular mesangial cells, endothelial cells, and podocytes express TLR1-6 [25]. Systemic exposure to TLR2, -3, and -4 agonists aggravates glomerular injury by directly activating these cell types which enhances glomerular inflammation and injury [26–29]. This process includes the recognition of endogenous danger signals, for example, the releases from dying cells [16]. Dying glomerular cells activate glomerular mesangial cells rather via TLR2/MyD88 signalling than TLR4/TRIF [30].

The role of HA in TLR signalling in mesangial cells was the topic of our investigation. According to previous reports from other cell types [3, 4, 12], it was to be clarified whether TLR2 and/or TLR4 are directly stimulated. Up to 3000 Da endotoxin-free HA was available. Further sizes of the molecule could be produced by hyaluronidase directly in the cell culture, since mesangial cells produce HA [31]. In this cell type, our data disprove the concept of direct ligation and favor HA as a regulator of TLR signalling by creating a barrier for receptors accessibility.

## 2. Methods

Primary mesangial cells (pMC) were isolated from C57/BL 6 mice including mice homocygotic deficient for Tlr2 or Tlr4 (pMC/TLR2^−/−^ and pMC/TLR4^−/−^) as described in detail elsewhere [30], stored in liquid nitrogen, and provided for the study. For the preparation of primary MC cells (pMC), capsule and medulla of the kidney were removed and the renal cortices were diced in cold phosphate buffered saline (PBS) and sequently passed through a series of stainless steel sieves (150, 103, 63, 50, and 45 *μ*m) and treated with a 1 mg/mL solution of type IV collagenase (Worthington, Lakewood, NY) for 15 minutes at 37°C. Finally, the digested glomeruli were seeded into 6-well plates with RPMI 1640 containing 20% fetal calf serum, 1% insulin, transferrin, and selenium (Roche, Mannheim, Germany). After five passages, >99% of pMC were positive for smooth muscle actin and >99% were negative for cytokeratin 18 (remark: in this study, the importance of hyaluronan on the cell surface turned to be superior to the distinct cell type). Cells were cultured in DMEM-glutamate-I + 10% fetal bovine serum + 1% penicillin/streptomycin at 37°C and 5% CO_2_.

50,000–100,000 cells were seeded in 24-well plates (TTP-Technologie, Trasadingen, Switzerland) and incubated at 37°C and 5% CO_2_ until the floor of the well was covered.

After washing with DPBS (PAN-BIOTECH GmbH, Aidenbach, Germany) the cells were incubated for 12–18 hours at 37°C and 5% CO_2_. The knockout status of the pMC/TLR2^−/−^ and pMC/TLR4^−/−^ cells was confirmed by real-time qPCR and by stimulation with ultrapure LPS (Invivogen; San Diego, CA, LPS-EB Ultrapure Cat.# tlrl-pelps) and PAM3CysSK4 (Invivogen; Pam3CSK4 Cat.# tlrl-pms). Cells were also incubated with HA < 1500 Da (lton) and 1500 Da to 3000 Da (Enzo Life Sciences; Cat.# ALX-580-004-C250; Cat.# ALX-580-005-C250) up to 50 *μ*g/mL, and HA (50 *μ*g/mL) together with LPS and PAM3CysSK4. In the next step, HYAL (Sigma-Aldrich, 750–3000 units/mg solid, Cat.#H4272; in M2 Cat.#M7167) with different concentrations (50 *μ*g/mL, 300 *μ*g/mL, and 1 mg/mL) was coincubated with 50 ng/mL of LPS and PAM3CysSK4. Then different concentrations of PAM3CysSK4 (1 ng/mL, 5 ng/mL, and 10 ng/mL) and LPS (10 ng/mL, 100 ng/mL, and 1 *μ*g/mL) were incubated with and without HYAL (1 mg/mL). Subsequently, PAM3CysSK4 and LPS (100 ng/mL) were co-incubated with and without HYAL (1 mg/mL) and with HYAL (1 mg/mL) plus heparin (4167 U/mL, Heparin-Natrium-5000-ratiopharm 5000 i.U./0,2 mL; ratiopharm GmbH) and its 1 : 10 and 1 : 100-dlutions as wells as enoxaparin (16,7 mg/mL, Clexane 40 mg/0,4 mL; Sanofi-Aventis GmbH] and its 1 : 10 and 1 : 100-dilutions. Active HYAL (1 mg/mL) was tested on both cell types, while it was tested on pMC/TLR2^−/−^ for HYAL inactivated at 95°C for 60 min with and without polymyxin B (PMB). Further studies were performed on pMC/TLR4^−/−^ cells with HYAL in different concentrations (50 *μ*g/mL, 300 *μ*g/mL, and 1 mg/mL) together with flagellin (TLR5-ligand, cell surface receptor, Invivogen; FLA-ST Ultrapure Cat.# tlrl-flic-10) in a concentration of 200 mg/mL, followed by an incubation of flagellin in a concentration of up to 1 *μ*g/mL and poly(I:C) (TLR3-ligand, intracellular receptor) (Invivogen; Poly(I:C) Cat.# tlrl-pic-5) in a concentration of up to 10 *μ*g/mL with and without HYAL (1 mg/mL). IL- (interleukin-)6 expression, examined in the supernatant, served as a marker of TLR activation, using the IL-6-ELISA (BD Biosciences, Cat.#555240) and NUNC-immunoplates (NUNC A/S, Roskilde, Denmark). The basis for the examination of the supernatant with an IL-6-ELISA was the study of Patole et al. [25]. Concentrations were used based on the studies in the literature [12, 32–37].

### 2.1. Microscopy

Finally, mesangial cells (pMC/TLR2^−/−^) were plated on a 6-well-plate and allowed to grow (37°C and 5% CO_2_) until the floor was partly covered. The supernatant was removed and a line drawn with (+)-*α*-tocopherol in soy-oil [Sigma-Aldrich, Cat.# T3634-25G]. On one of the 2 created separate regions, 20 *μ*L of HYAL (10 mg/mL) was dropped. Both areas were allowed to dry out and referred to microscopy (microscopy and documentation: Leica DMIL-Mikroscope, Leica Microsystems Heidelberg GmbH, Heidelberg, Germany) and visualising system ProgRes Capture Pro 2.1 (Jenoptik AG, Jena, Germany) on an iMAC-Computer [Apple Inc, 1 Infinite Loop Cupertino, CA, USA] with MacOS (X) at a 400-fold magnification).

### 2.2. Statistics

The statistical analysis was performed using “GraphPad Prism 5” (GraphPad Software, Inc., 2236 Avenida de la Playa, La Jolla, CA, USA) and Excel (Microsoft, Redmond, WA, USA). The mean and the standard deviation of the mean were calculated. We used unpaired Student's *t*-test for all comparisons. The level of significance was 95% and the *P* value was classified as not significant *P* > 0.05 and significant *P* ≤ 0.05, with grades of *–****, *P ≤ 0.05, **P < 0.01, ***P < 0.001, and ****P < 0.0001. “GraphPad Prism 5” was also used for illustration.

## 3. Results

### 3.1. Hyaluronan < 3000 Da Does Not Activate Mesangial Cells

**Figure 1:**
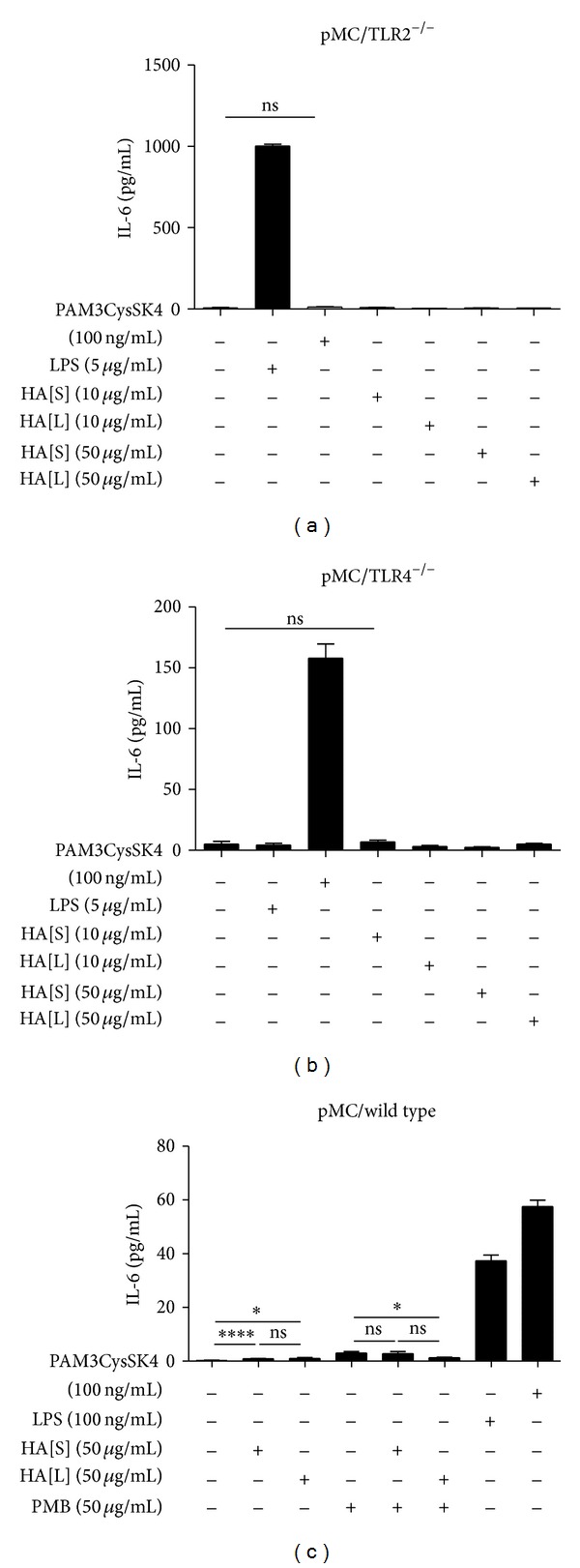
Mesangial cells do not produce IL-6 upon stimulation with hyaluronan. TLR2-deficient (a), TLR4-deficient (b), and wild type (c) mesangial cells were exposed to hyaluronan of different sizes and concentrations as indicated. TLR2 and TLR4 agonists were used as positive controls for IL-6 induction. Additionally, wild type cells incubated with hyaluronan plus polymyxin B to exclude the influence of a random LPS-contamination. HA[S]: hyaluronan < 1500 Da; HA[L]: hyaluronan 1500 Da–3000 Da; PMB: polymyxin B; data are means ± SD, ns: not significant, *P ≤ 0.05–****P < 0.0001.

Both types of knockout mesangial cells (pMC/TLR2^−/−^ cells and pMC/TLR4^−/−^ cells) were incubated with PAM3CysSK4 (100 ng/mL), LPS (5 *μ*g/mL), and HA < 1500 Da (10 *μ*g/mL and 50 *μ*g/mL) and HA 1500–3000 Da (10 *μ*g/mL and 50 *μ*g/mL). In mesangial wild type cells, a setting of HA with polymyxin B was added. While, in this experiment with knockout cells, 2 wells of a 24-well plate were incubated for every setting and duplicates were taken from each well for IL-6 ELISA; for all of the other experiments, 3 wells were incubated and duplicates were taken. pMC/TLR2^−/−^ cells produced IL-6 upon LPS but not upon PAM3CysSK4 exposure (Figure 1(a)). The opposite results were obtained with pMC/TLR4^−/−^ cells (Figure 1(b)), which confirmed the knockout status of the cells as well as the intact signalling cascade to produce IL-6. HA < 3000 Da, however, did not induce IL-6 release in both cell types up to a concentration of 50 *μ*g/mL (Figure 1). Also, in wild type cells with and without polymyxin B, the level of IL-6 production remained the same with and without HA < 3000 Da. Thus, HA < 3000 Da does not directly stimulate mesangial cells.

### 3.2. Hyaluronan < 3000 Da Does Not Link TLR2 and -4 Agonists to Any other than Their Specific Ligand with Subsequent IL-6 Expression

**Figure 2:**
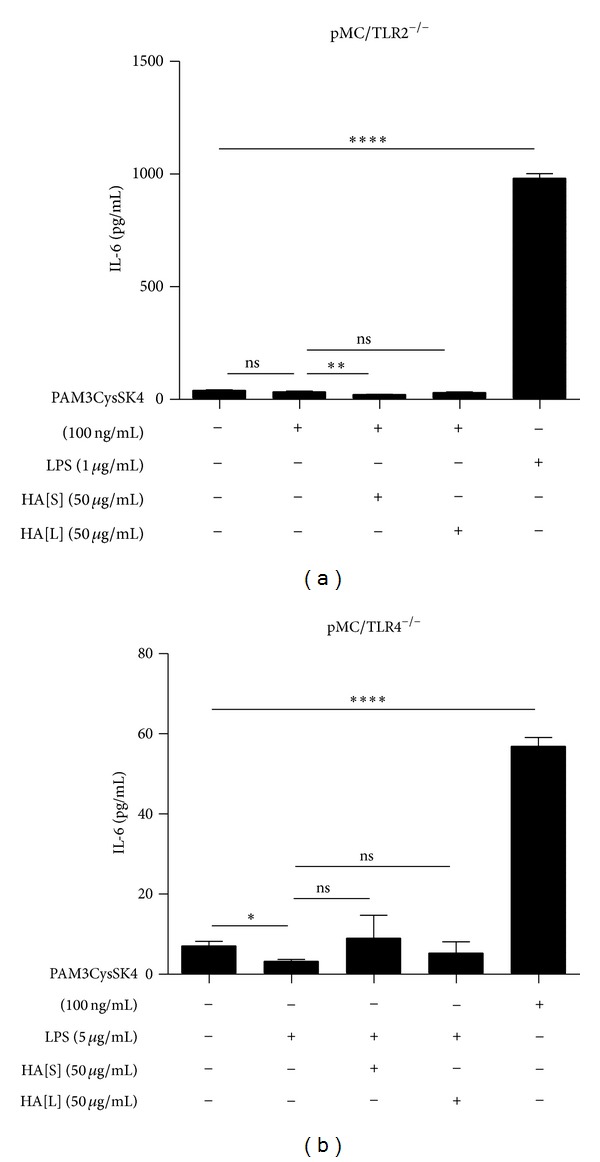
Evaluation of functional relevant unspecific linking capacity of hyaluronan. It was tested, if HA could link PAM3CysSK4 or LPS to any other than its specific TLR. Therefore HA was incubated with PAM3CysSK4 (TLR2 ligand) on TLR2-deficient cells (a), with LPS as a control. The double incubation could not induce an IL-6 expression. Analogously, HA was incubated together with LPS (TLR4 ligand) on TLR4-deficient cells (b), with PAM3CysSK4 as a control. Also, here, the double incubation did not induce an IL-6 expression. Conclusively, an unspecific linking function of HA could not be detected in this setting. HA[S]: hyaluronan < 1500 Da; HA[L]: hyaluronan 1500 Da–3000 Da; data are means ± SD, ns: not significant, *P ≤ 0.05–****P < 0.0001.

Next, we tested whether adding HA to the medium would affect TLR signalling in mesangial cells. LPS (on pMC/TLR2^−/−^ cells) (Figure 2(a)) and PAM3CysSK4 (on pMC/TLR4^−/−^) (Figure 2(b)), respectively, were incubated with and without 50 *μ*g/mL HA < 1500 Da and HA 1500 Da−3000 Da. Adding HA did not significantly increase IL-6 release. These data suggest that HA < 3000 Da stimulation does not modulate TLR2 or TLR4 signalling and that HA does not link TLR2 and TLR4 agonists to any other pattern recognition receptor. HA does not act as an unspecific linker molecule.

### 3.3. Hyaluronan Breakdown in the Cell Culture Does Not Directly Activate Mesangial Cells

**Figure 3:**
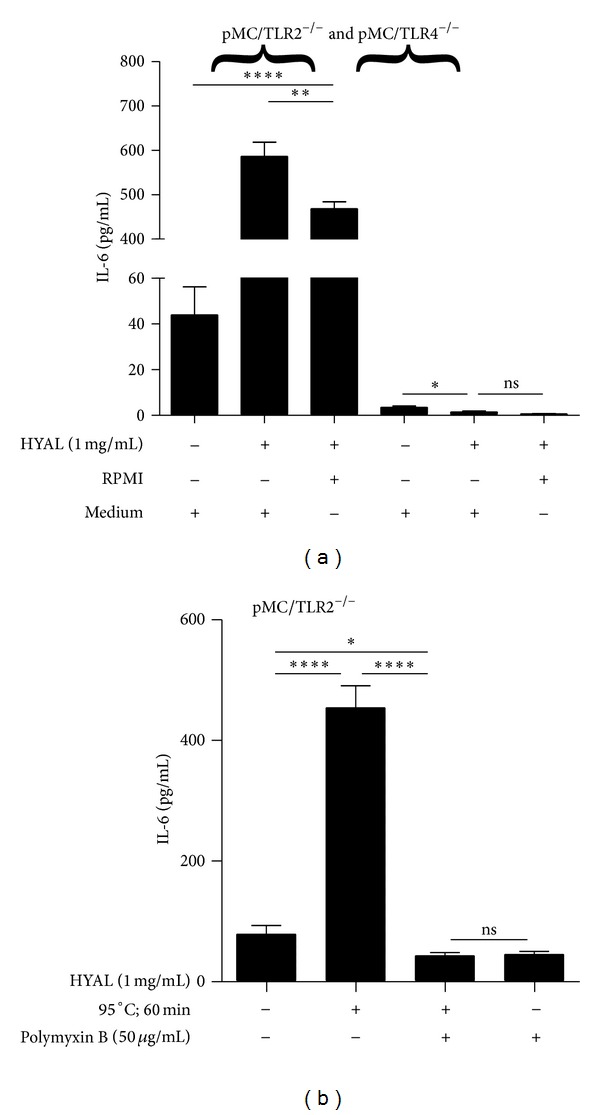
Hyaluronidase alone does not activate mesangial cells in TLR4-deficient mesangial cells. pMC/TLR2^−/−^ and pMC/TLR4^−/−^ cells were incubated with HYAL as indicated and IL-6 production was measured by ELISA. HYAL, which produced hyaluronan fragments of different sizes, induced IL-6 production only in pMC/TLR2^−/−^ cells (a). This effect was due to an LPS contamination of the as shown by enzyme inactivation (95°C, 60 min), change of the medium, and blocking of LPS-influence with polymyxin B (b). HYAL: hyaluronidase; PMB: polymyxin B; RPMI: nutritive medium; medium was exchanged against RPMI 1640; data are means ± SD, ns: not significant, *P ≤ 0.05–****P < 0.0001.

HA breakdown products have been reported to elicit TLR agonistic activity in immune cells, for example, upon digesting HA with HYAL into smaller fragments [3]. Therefore, we treated mesangial cells with HYAL to see whether the HA produced by the mesangial cells would turn into a danger signal upon degradation into smaller elements. HYAL induced IL-6 secretion in pMC/TLR2^−/−^ cells, while it did not in pMC/TLR4^−/−^ cells (Figure 3(a)). The IL-6 response in pMC/TLR2^−/−^ cells persisted after heat inactivation of HYAL (1 mg/mL) at 95°C for 60 minutes but was abrogated by 50 *μ*g/mL polymyxin B which binds LPS [37] (Figure 3(b)). Obviously, an endotoxin contamination of the HYAL preparation was responsible for this stimulatory effect in cells that had TLR4 to recognize it. From the results in the TLR4-deficient cells, we can conclude that degrading the macromolar external HA sheath of mesangial cells with HYAL does not generate components that can induce IL-6 production. This excludes a TLR agonistic activity of low molecular weight HA.

### 3.4. Hyaluronidase Degrades the Extracellular Matrix of Mesangial Cells

**Figure 4:**
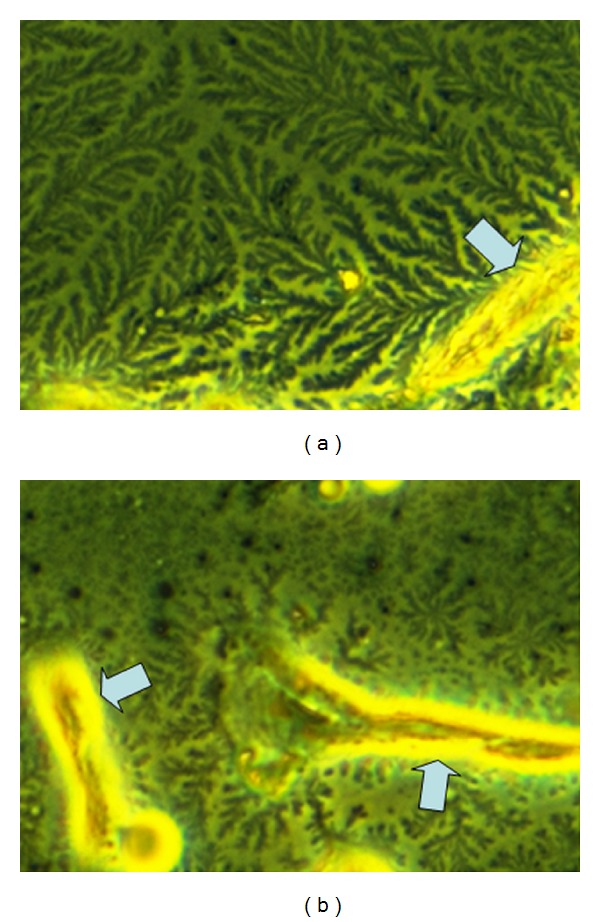
Illustration of digestion of mesangial cell-related extracellular matrix with hyaluronidase. Mesangial cells were incubated without (a) and with (b) hyaluronidase. Branch-like structures on the cell surface (a) are at least containing hyaluronan, since they are cleaved by hyaluronidase (b) to minor fragments. While high molecular hyaluronan binds water to form a jelly, low molecular hyaluronan forms a liquid solution. So the branches in (a) illustrate the jelly barrier on the cell surface. (a) Mesangial cells form branch-like structures around the outer aspect of the cells (arrow). (b) Hyaluronidase digestion leads to rapid fragmentation of this matrix. Original magnification ×400.

pMC/TLR4^−/−^ cells were incubated with and without 1 mg/mL HYAL. Then, the cell layer was allowed to dry to unmask extracellular matrix by phase contrast microscopy (Figures 4(a) and 4(b)). In the absence of HYAL, the formation of structures reminding on branches of a tree on the surface of the cells could be observed (Figure 4(a)). Upon HYAL digestion, these branches were broken down into smaller pieces, which could be observed on the cell surface, while isolated small fragments of these branches were found without any cell contact (Figure 4(b)). Thus, HYAL breaks down the extracellular matrix around mesangial cells.

### 3.5. Hyaluronidase Increases the Effect of Agonists to TLR2, TLR3, TLR4, and TLR5

**Figure 5:**
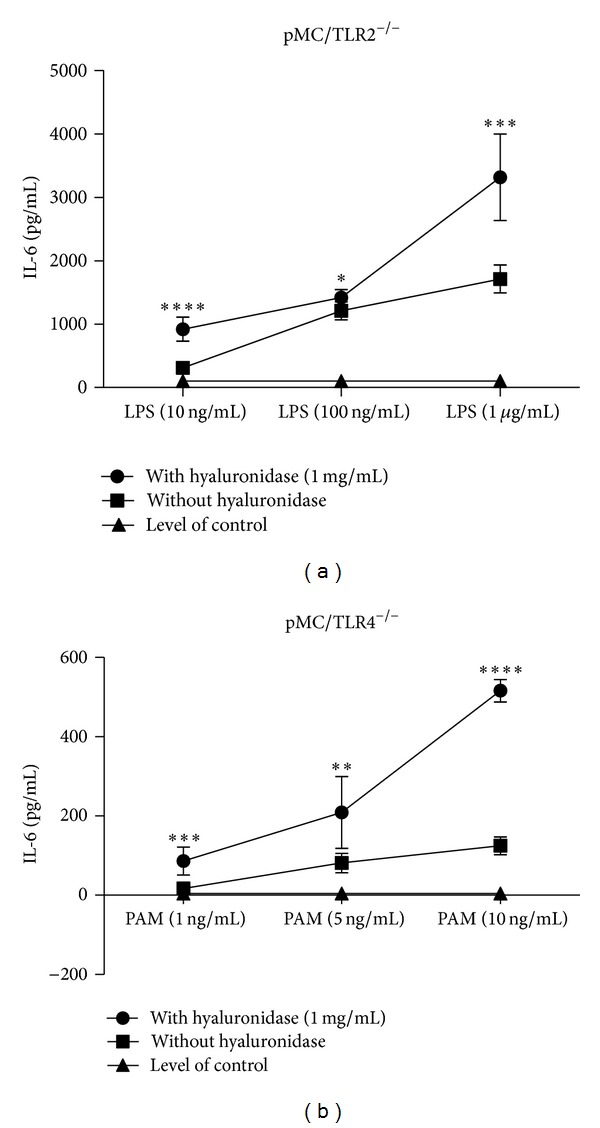
Effect of hyaluronidase on the specific stimulation of TLR2 and TLR4. Both cell types were incubated with different concentrations of specific TLR ligands ((a) TLR2 and (b) TLR4) with and without hyaluronidase to demonstrate the effect of hyaluronidase on TLR-stimulation. PAM: PAM3CysSK4; HYAL: hyaluronidase; data are means ± SD, ns: not significant, *P ≤ 0.05–****P < 0.0001.

**Figure 6:**
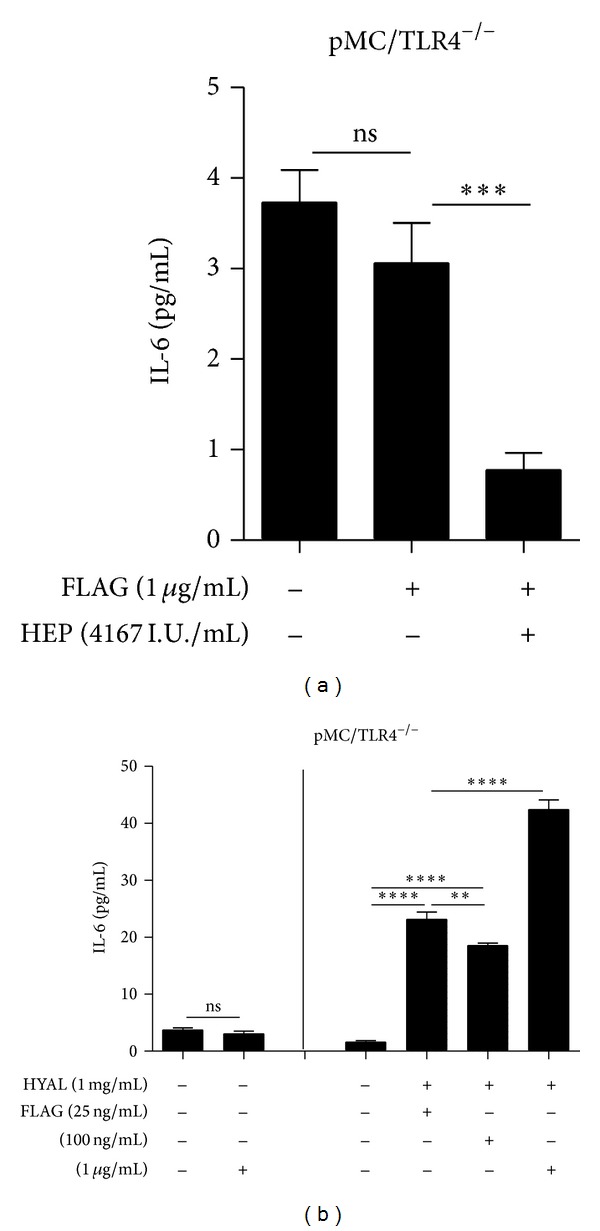
Effect of heparin and hyaluronidase on the specific stimulation of TLR5. pMC/TLR4^−/−^ (cells deficient of TLR4) were incubated with (a) TLR5 ligand with and without heparin and (b) different concentrations of a specific TLR5 ligand with and without hyaluronidase to demonstrate the effects of heparin and hyaluronidase on TLR stimulation. FLAG: flagellin; HYAL: hyaluronidase; HEP: heparin; data are means ± SD, ns: not significant, *P ≤ 0.05–****P < 0.0001.

**Figure 7:**
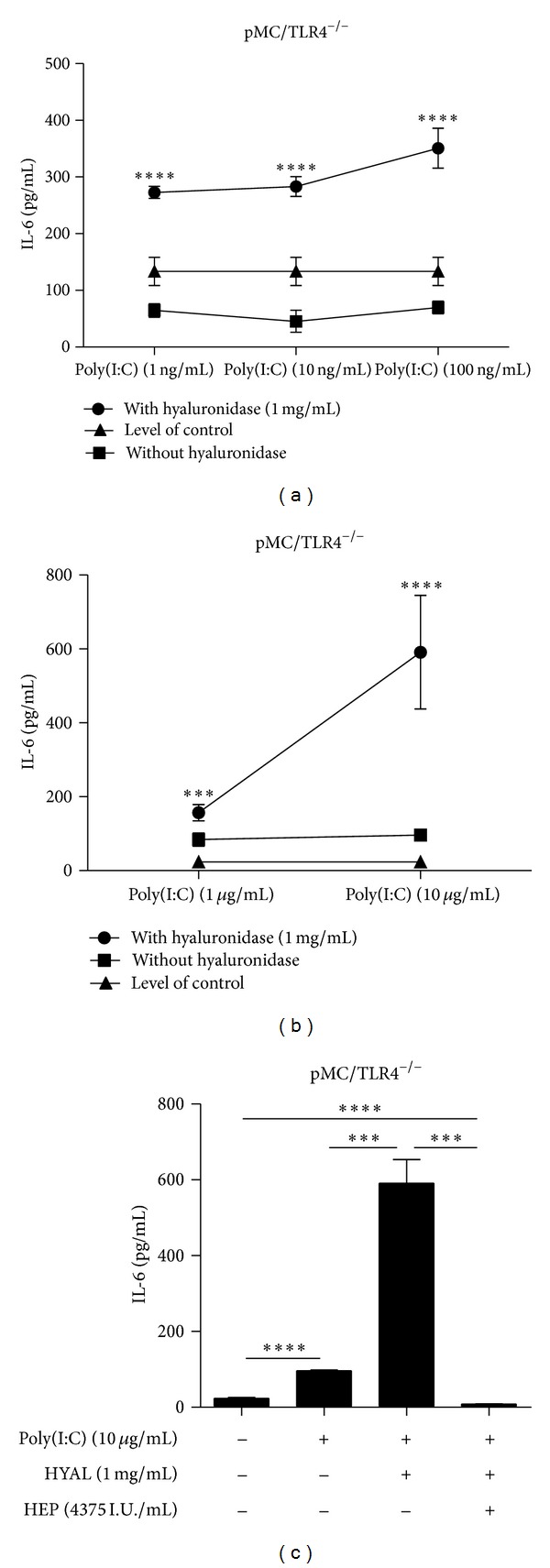
Effect of hyaluronidase and heparin on the specific stimulation of TLR3. (a, b) pMC/TLR4^−/−^ cells types were incubated with different concentrations of a specific TLR3 ligand with and without hyaluronidase to demonstrate the effect of hyaluronidase on TLR stimulation. (c) Finally, the influence of heparin on the coincubation with hyaluronidase and TLR3 ligand was tested. Poly: Poly(I:C); HYAL: hyaluronidase; HEP: heparin; data are means ± SD, ns: not significant, *P ≤ 0.05–****P < 0.0001.

HA forms a jelly shield around cells which limits the accessibility of nutrients and potential danger signals to the cell surface. Therefore, we next evaluated whether HA degradation affected TLR signalling induced by specific TLR agonists. Mesangial cells mainly express TLR1-6 [24], of which specific agonists are available for TLR2, TLR3, TLR4, and TLR5. In both cell types, 1 mg/mL HYAL caused an increase of IL-6 release upon stimulation with the ligands for TLR2 (1, 5, or 10 ng/mL PAM3CysSK4) and TLR4 (10, 100, and 1000 ng/mL LPS) together with HYAL (Figures 5(a) and 5(b)). The same applied to stimulation with ligands for TLR5 (Figures 6(a) and 6(b)) and TLR3 (Figures 7(a), 7(b), and 7(c)). The agonists for TLR2, TLR3, and TLR5 were tested only in pMC/TLR4^−/−^ cells to avoid the impact of LPS contamination of the HYAL preparation. Thus, HA degradation with HYAL, a process known to reduce the viscosity and the thickness of the HA shield around cells, increased the stimulatory capacity of agonists to TLR2, TLR3, TLR4, and TLR5.

### 3.6. Heparin Suppresses the Agonistic Effect of TLRs on Mesangial Cells

**Figure 8:**
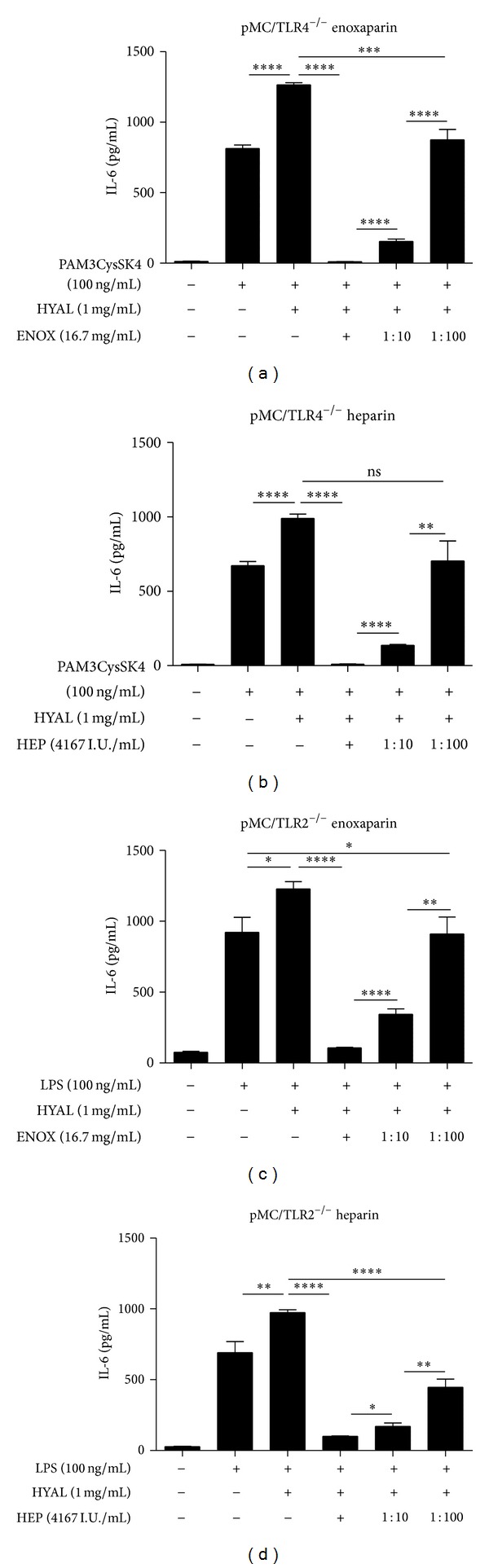
Influence of different concentrations of heparin on specific stimulation of TLR2 and TLR4. Both cell types were coincubated TLR ligands with and without hyaluronidase of constant concentrations. Heparins of different sizes were tested on both cell types coincubated with the TLR ligand and hyaluronidase to demonstrate the inhibitory effect of heparin on IL-6 expression. (a) enoxaparin on pMC/TLR4^−/−^ (TLR4-deficient cells), (b) heparin on pMC/TLR4^−/−^, (c) enoxaparin on pMC/TLR2^−/−^ (TLR2-deficient cells), and (d) heparin on pMC/TLR2^−/−^. PAM: PAM3CysSK4; HYAL: hyaluronidase; ENOX: enoxaparin; HEP: heparin; data are means ± SD, ns: not significant, *P ≤ 0.05–****P < 0.0001.

Heparin, a substance with immunoregulatory properties [38], is another extracellular glycosaminoglycan polymer and was reported to be an antagonist of hyaluronidase [39]. The observed heparin effect exceeded the mere compensation of the hyaluronidase effect. IL-6 expression upon stimulation with poly(I:C) RNA (10 *μ*g/mL) plus HYAL (1 mg/mL) plus heparin (4375 I.U/mL) was significantly less than that for poly(I:C) RNA alone (Figure 7(c)). This effect was confirmed upon LPS (100 ng/mL) and PAM3CysSK4 (100 ng/mL) stimulation instead of poly(I:C) RNA. It is of note that high molecular weight heparin (4167 I.U/mL) and low molecular weight heparin (enoxaparin) (16.7 mg/mL) had the same effect to compensate degraded HA, both of them in a dose-dependent manner because decreasing heparin (1 : 10; 1 : 100) concentrations also decreased the inhibitory effect (Figures 8(a), 8(b), 8(c), and 8(d)). Thus, heparin inhibits the response to TLR stimulation upon HA degradation in a dose-dependent manner. Hence, we questioned whether macromolecular heparin could compensate HA degradation with HYAL in terms of TLR signalling on mesangial cells.

## 4. Discussion

Our initial hypothesis of HA being a TLR2 and/or TLR4 agonist was based on previous reports in other cell types. The concept was disproved for mesangial cells also by using TLR2- or TLR4-deficient cells which allowed us to eliminate the problem of potential TLR agonist contaminations of the preparations used. Termeer et al. reported no further stimulatory effect of HA beyond 25 *μ*g/mL, so that we considered the use of 50 *μ*g/mL a sufficient dose [12]. At this concentration, HA < 3000 Da had neither an intrinsic nor linked ligands unspecifically to another receptor, in the present study. HA consists of D-glucuronate and D-N-acetylglucosamine in an alternating sequence [3], lacking additional binding sites in larger molecules. The LPS binding of TLR4 [40] and between TLR2 and its ligand lipoprotein [41] is based on their lipophilic binding sites, and the binding activates the signalling cascade. HA as a hydrophilic molecule should not be able to bind directly to any of these lipophilic receptors, at least not to the same binding sites as the named ligands. Thus, HA was excluded as an endogenous danger signalling molecule that can activate mesangial cells (via TLR2 and TLR4).

HA is ubiquitously distributed throughout the organism [4] and the backbone of a jelly shield around the cell [5], including mesangial cells [31, 42]. Mesangial cells incubation with HYAL broke down the extracellular matrix, especially, the HA component to produce HA fragments of different sizes. This did not cause an answer in TLR4-deficient mesangial cells, further excluding a stimulatory property of HA fragments. HYAL itself induced a response in TLR4 competent cells, which we related to an LPS contamination. This we concluded from its persistent immunostimulatory effect after heat inactivation and from coincubation with polymyxin B, which neutralized LPS [37]. Obviously, HA itself does not activate TLR signalling but regulates the accessibility of the receptors via a barrier mechanism. This conclusion was supported by the finding that ligands of various TLRs (PAM3CysSK4, poly I:C RNA and flagellin) increased the IL-6 response, when combined with HYAL compared to the stimulation without HYAL in TLR4-deficient cells. HYAL and thereby the HA-fragments of any produced size itself did not have a stimulatory effect on the cells. So the stimulatory effect of HYAL in combination with TLR2-/TLR3-/TLR5-ligand could only be addressed to the ligand. The increased response to LPS in TLR4 competent cells did not allow a differentiation between the effect of the enzyme and the LPS contamination.

Breaking down the HA barrier gives access to the receptors or adhesion molecules. Due to the reduced water binding capacity, the thickness and viscosity of the jelly-like coat are decreased. The effect of venoms, which contain HYAL [43] explains the edema, since the low-molecular weight HA has a lower binding capacity for water [7]. The inflammatory reaction is the result of an increased accessibility of the receptors. Enzymatic degradation of HA is supposed to occur quickly which explains the quick development of the clinical symptoms. The access to an increased number of receptors by one-step degradation of the jelly-like coat is supposed to occur quicker than by new synthesis and consecutive integration of receptors into the membrane. Hence, the amount of HA on the surface of the cells may play a role in the pathogenesis of many diseases with an immunologic background [3]. HA synthesis and breakdown and the amount of HA receptors are of interest in this context. Increased binding of HA by CD44 on the cell surface leads to a reduced immune response on LPS stimulation of TLR4 [44]. The induction of HA of mesangial cells in lupus-like nephritis can be interpreted as a protective mechanism to reduce the inflammatory activity due to external stimulation [42].

Heparin suppressed the IL-6 response on LPS, PAM3CysSK4, and poly(I:C) combined with HA, which exceeded the described inhibitory effect on HYAL [39]. This suppressing effect, observed for high-molecular weight heparin (heparin) and low-molecular weight heparin (enoxaparin), declined with increasing dilutions of heparin, when examined in coincubation with LPS, PAM3CysSK4, and HYAL on pMC/TLR2^−/−^ and pMC/TLR4^−/−^ cells. These results raised the idea of a barrier effect also of heparin, like HA, a member of the glycosaminoglycan-family.

In summary, the extracellular macromolecule HA is the matrix of a jelly-like barrier around mesangial cells. HA breakdown, for example, by HYAL, produces HA fragments, which in contrast to previous reports do not induce TLR signalling, at least not in mesangial cells. However, HA breakdown decreases the thickness and viscosity of the jelly-like barrier, which increases the TLR accessibility for TLR ligands. Hence, we conclude that HA is not a TLR-ligand but a regulator of TLR signalling, at least on glomerular mesangial cells.

## 5. Conclusions

On the basis of the presented results, hyaluronan does not stimulate TLR receptors but regulates their accessibility, at least in mesangial cells. Hyaluronan is the backbone of a jelly-like barrier on the cell surface. The thickness and viscosity of the jelly-like barrier regulate the accessibility of the Toll-like receptors for at least the examined ligands.

Heparin has an effect beyond the blocking of hyaluronidase and reduces the answer to TLR stimulation.
